# Salts of 2-amino-5-iodo­pyridinium

**DOI:** 10.1107/S2056989024010259

**Published:** 2024-10-31

**Authors:** Benjamin A. Mukda, Diane A. Dickie, Mark M. Turnbull

**Affiliations:** a Carlson School of Chemistry and Biochemistry, Clark University, 950 Main St., Worcester, MA 01610, USA; b Dept. of Chemistry, University of Virginia, McCormack Rd., Charlottesville, VA 22904, USA; Venezuelan Institute of Scientific Research, Venezuela

**Keywords:** crystal structure, 2-amino-5-iodo­pyridine, Co(II)

## Abstract

Reaction of 2-amino-5-iodo­pyridine (5IAP) with concentrated HBr at room temperature yielded 2-amino-5-iodo­pyridinium bromide, C_5_H_6_IN_2_^+^·Br^−^ or (5IAPH)Br. The complex formed pale-yellow crystals, which exhibit significant hydrogen bonding between the amino and pyridinium N—H donors and bromide ion acceptors. Halogen bonding is also observed. Similarly, reaction of 5IAP with cobalt(II) chloride in mixed HCl/HBr in 1-propanol yielded 2-amino-5-iodo­pyridinium (2-amino-5-iodo­pyridine-κ*N*^1^)bromido/chlorido­(0.51/2.48)cobalt(II), (C_5_H_6_IN_2_)[CoBr_0.51_Cl_2.48_(C_5_H_5_IN_2_)] or (5-IAPH)[(5IAP)CoCl_2.48_Br_0.51_], as blue block-shaped crystals. Two of the three halide positions exhibit mixed occupancy [Cl/Br = 0.797 (5):0.203 (5) and 0.689 (6):0.311 (6)], while the third position is occupied solely by a chloride ion. Extensive hydrogen and halogen bonding is observed.

## Abstract 2

Compound **1** is the anhydrous form of the known crystal 2-amino-5-iodo­pyridinium bromide monohydrate and crystallizes in layers inclined ∼40° to the *ab* face. Hydrogen bonding between the amino and pyridinium ions to the bromide ion acceptor stabilizes the layers. Compound **2** is a salt of 2-amino-5-iodo­pyridium and a trihalidocobaltate(II) ion with one coordinated 2-amino-5-iodo­pyridine ligand. The halide ions are mixed Cl/Br with differing occupancies.

## 1. Chemical context

The effects of randomness have been of particular inter­est in physics and chemistry. In particular, they have been considered regarding quantum information (Khrennikov, 2016 ▸), band theory (Coey *et al.*, 2005 ▸) and perturbation of the crystal lattice (Mackenzie, 1964 ▸; Anderson, 1958 ▸). With respect to magnetism, studies have looked at the relationship between randomness and spin glasses (Toulouse, 1986 ▸), amorphous magnets (Coey, 1978 ▸) and valence-bond solids (Kimchi *et al.*, 2018 ▸).

Superexchange in magnetic systems can be studied through the production of families of closely related compounds where small changes in the structure can be correlated with their effects on the magnetic properties of the materials. We have looked at the production of such complexes, especially those based upon salts of subsituted 2-amino­pyridine for some time (Araujo-Martinez *et al.*, 2023 ▸; Coffey *et al.*, 2000 ▸; Landee *et al.*, 2001 ▸; Woodward *et al.*, 2002 ▸). One such compound, 2-amino-5-iodo­pyridine, has been involved in the production of a magnetic ladder (Landee *et al.*, 2001 ▸) and a family of Cu^II^ halides complexes (Huynh *et al.*, 2023 ▸).

One difficulty in the studies of randomness in such materials is the introduction of randomness into an otherwise ordered system. Crystallization is intrinsically a self-purifying process and attempts to introduce randomness through introduction of dopants into a system may be frustrated by exclusion of the ‘impurity’ during crystallization (Fujiwara *et al.* 1995 ▸). We have recently discovered a system, based upon 2-amino-5-iodo­pyridine (5IAP), where randomness can be introduced to the system *via* introduction of a mixture of halide ions; (5IAPH)_2_[CoCl_4–*x*_Br_*x*_]·H_2_O (Mukda *et al.*, 2024 ▸) where 5IAPH is 2-amino-5-iodo­pyridinium. In the course of those investigations, we isolated the related compound (5IAPH)[(5IAP)CoCl_3–*x*_Br_*x*_] and here report its structure and the structure of the related salt (5IAPH)Br.
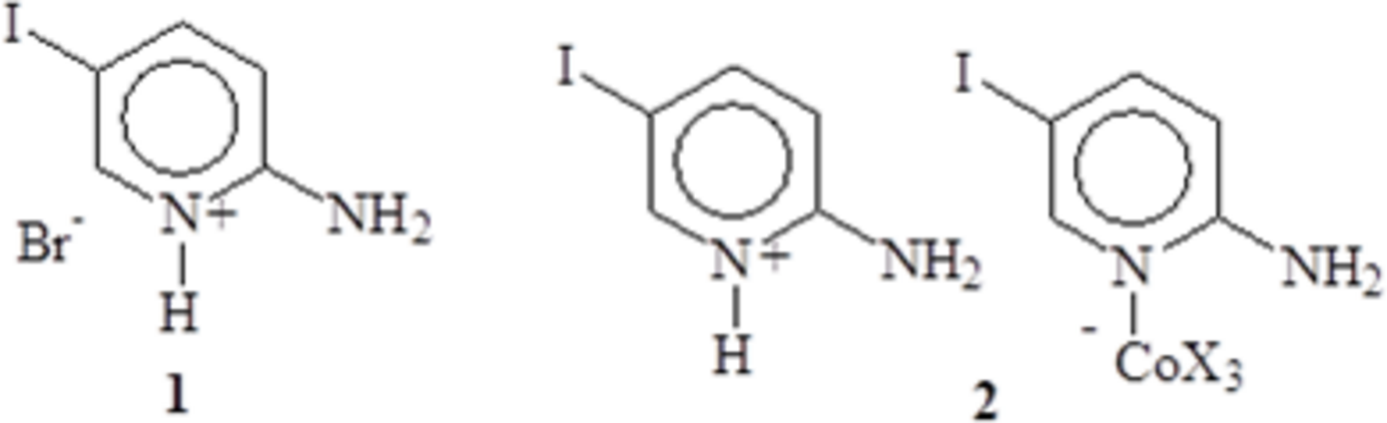


## 2. Structural commentary

**Figure 1:**
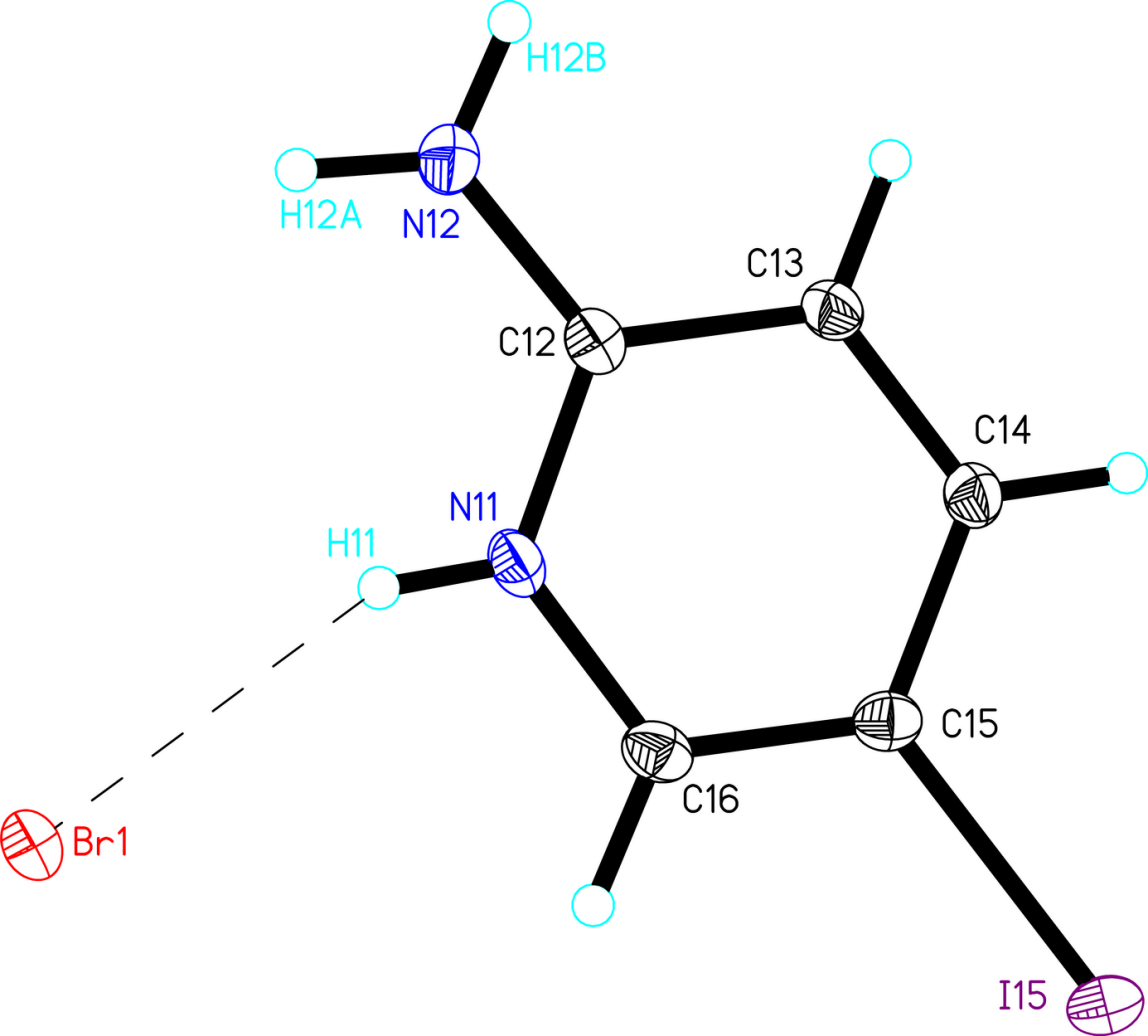
The asymmetric unit of **1** shown as 50% probability ellipsoids (hydrogen atoms are shown as spheres of arbitrary size). Only those hydrogen atoms whose positions were refined are labeled.

**Figure 2:**
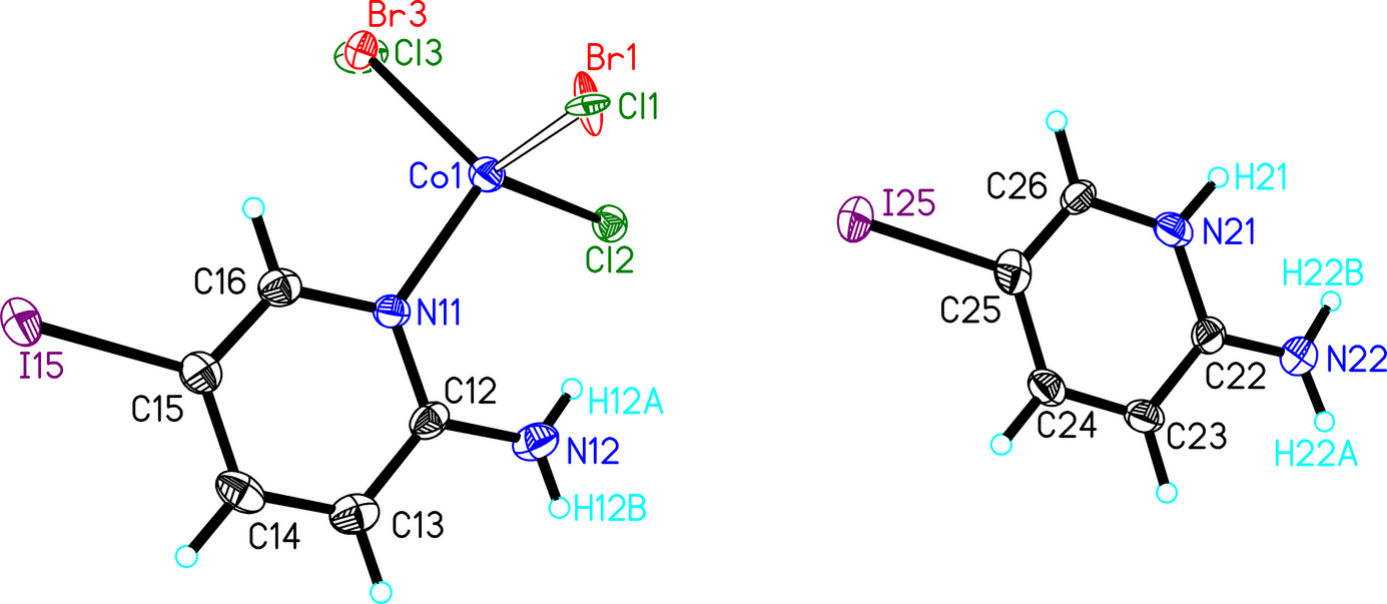
The asymmetric unit of **2** shown as 50% probability ellipsoids (hydrogen atoms are shown as spheres of arbitrary size). Only those hydrogen atoms whose positions were refined are labeled.

(5IAPH)Br (**1**) crystallized in the triclinic space group *P*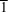 and comprises one 5IAPH cation and one bromide anion in the asymmetric unit (Fig. 1 ▸). The 5IAPH ring is planar (mean deviation of constituent atoms = 0.012 Å) with the amino substituent lying 0.070 (1) Å out of that plane. The iodine atom is displaced significantly further out of the plane, 0.199 (1) Å, toward the same face. The amino substituent deviates only slightly from *sp*^2^-hybridization [sum of ∠s = 360 (2)°]. The plane of the NH_2_ group is nearly co-planar with the 5IAPH ring [6.8 (15)°] as expected due to conjugation.

(5IAPH)[(5IAP)CoCl_3–*x*_Br_*x*_] (**2**) crystallized in the monoclinic space group *P*2_1_/*n*. The asymmetric unit is shown in Fig. 2 ▸ and comprises one 5IAPH cation and one [(5IAP)CoCl_3–*x*_Br_*x*_] anion. The 5IAPH cation is nearly identical to that observed in **1**, with a high degree of planarity in the ring (±0.007 Å), the sum of the angles about the amino nitro­gen atom being 359 (2)° and the amino group being nearly co-planar with the 5IAPH ring [deviation = 8.9 (16)°]. As with **1**, the amino nitro­gen atom [0.013 (5) Å] and iodine atom [0.086 (1) Å] are displaced slightly from the plane of the ring, again both toward the same face. The anionic unit comprises one Co^II^ ion with a 5IAP ring coordinated through the pyridine nitro­gen atom and three coordinated halide ions. The halide ions are mixed Cl/Br with refined occupancies of Cl1/Br1 [0.797 (5)/0.203 (5)] and Cl3/Br3 [0.689 (6)/0.311 (6)]. Attempts to refine the position of Cl2 as mixed Cl/Br resulted in an occupancy of Cl2 of 1.0 within error; no bromide ion was included in that position in the final refinement. The Co—*X* bond lengths are all similar (∼2.3 Å) regardless of halide ion (Table 1 ▸). The Co^II^ ion is only slightly distorted from tetra­hedral with bond angles ranging from 105.1 (3) to 116.9 (3)°. The 5IAP ring is comparable to the 5IAPH ring in terms of planarity (mean deviation = 0.006 Å) and displacement of N12 and I15 [0.09 (1) Å and 0.077 (1) Å, respectively]. The amino group is again planar, but inclined 17 (2)° relative to the 5IAP plane, likely to accommodate the intra­molecular N12—H12*A*⋯Cl2 hydrogen bond [*D*⋯*A* = 3.282 (6) Å; Table 3].

## 3. Supra­molecular features

**Figure 3:**
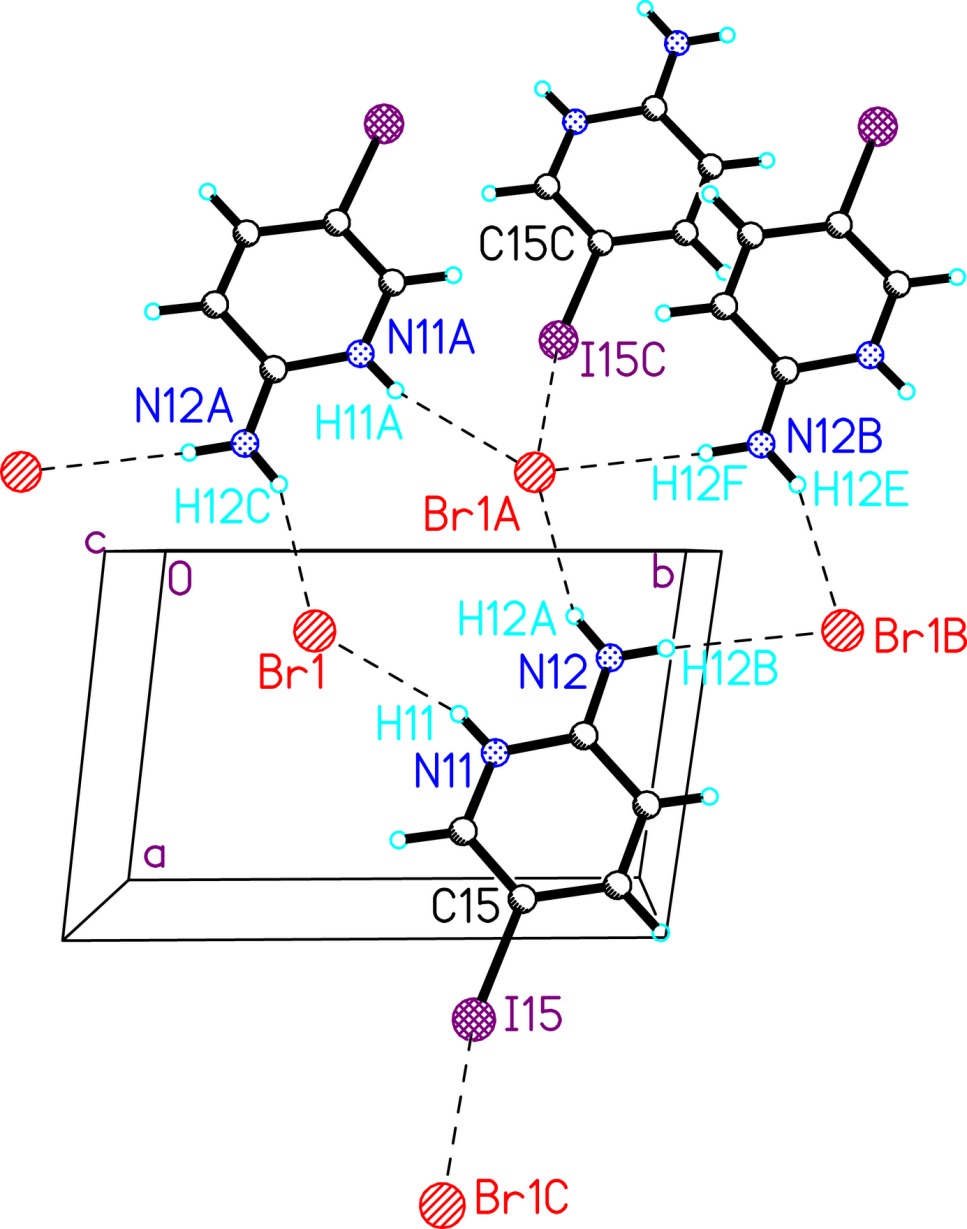
Halogen and hydrogen bonding in **1** (atoms are shown as spheres of arbitrary size). Dashed lines represent hydrogen and halogen bonds. Only those atoms involved in halogen or hydrogen bonding are labeled. Symmetry codes: Br1A = −*x*, 1 − *y*, −*z*; Br1B = *x*, *y* + 1 *z*; N12B = −*x*, 2 − *y*, −*z*; I15C = 1 − *x*, *y*, *z* − 1.

**Figure 4:**
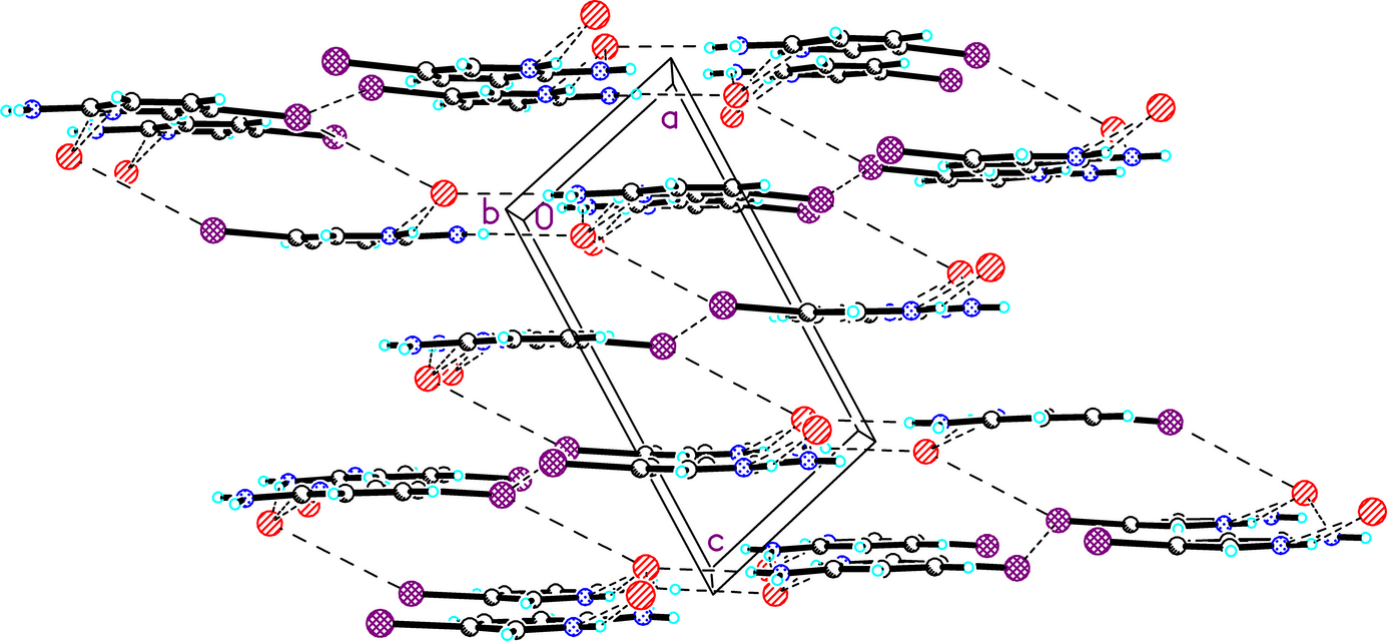
The structure of **1** viewed parallel to the *b* axis (atoms are shown as spheres of arbitrary size). Dashed lines represent hydrogen and halogen bonds.

Compound **1**. Extensive hydrogen (Table 2 ▸) and halogen bonding with the bromide ion as acceptor are present in the structure (Fig. 3 ▸). The hydrogen bonds are typical with *D*⋯*A* distances ranging from 3.2136 (12) to 3.4924 (13) Å and *D*—H⋯*A* angles of 150.8 (18) to 165.5 (18)°. Each bromide ion serves as an acceptor of three hydrogen bonds from the pyridinium N—H and both protons on the amino group. The latter generates inversion-related pairs of 5IAPH ions bridged by the bromide ions (Fig. 3 ▸). A Type II halogen bond is also present with parameters *d*_I15*C*⋯Br1_ = 3.88 (1) Å and ∠_C15—I15*C*⋯Br1_ = 154.6 (4)°. Further halogen bonding is observed in the packing structure (Fig. 4 ▸). Sheets of 5IAPH and bromide ions are linked parallel to the *a* axis by Type I halogen bonds between inversion-related iodine atoms; *d*_I15⋯I15*A*_ = 3.81 (1) Å and ∠_C15—I15⋯I15*A*_ = 130.6 (3)° [symmetry code: (A) 1 − *x*, 1 − *y*, 1 − *z*].

**Figure 5:**
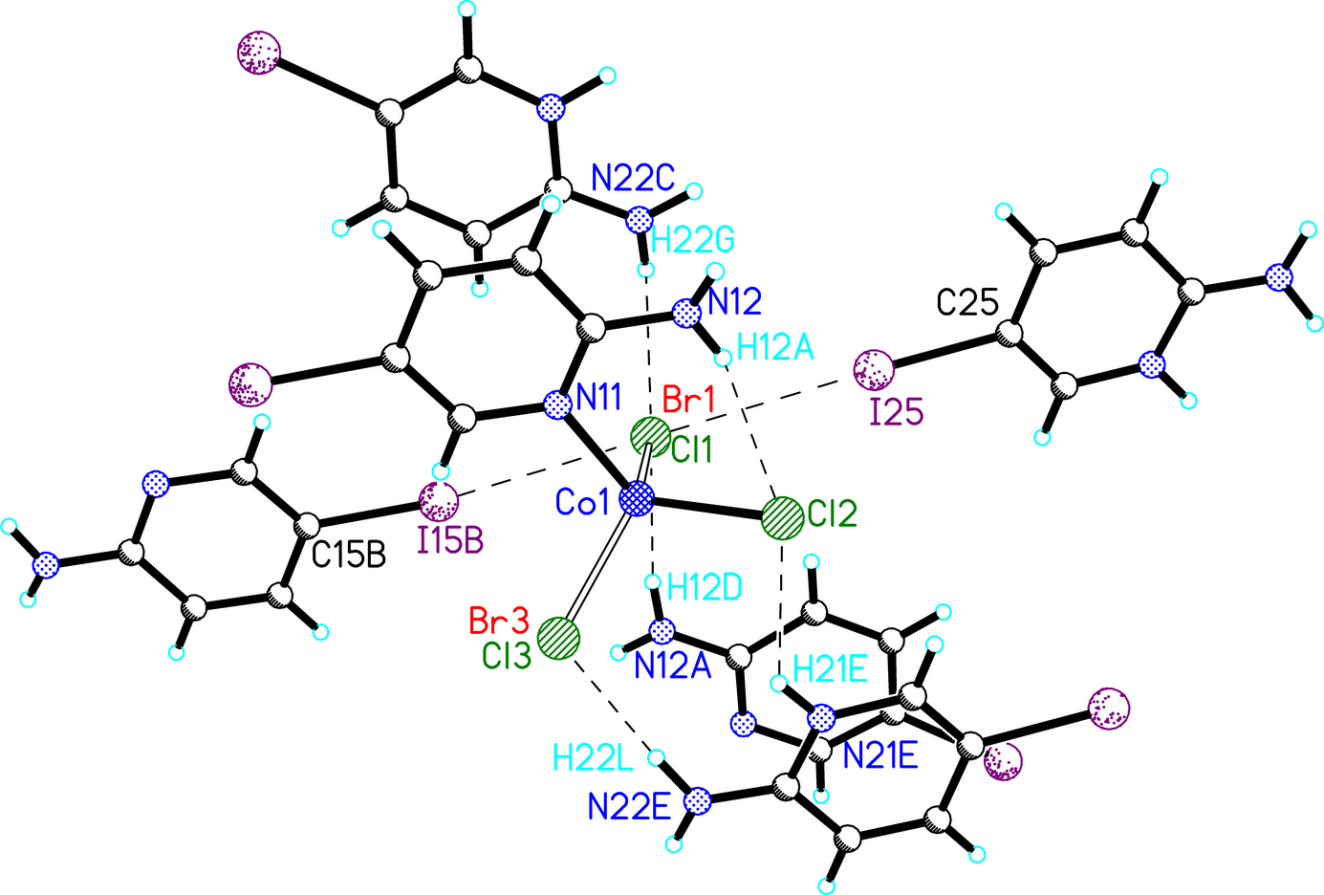
Halogen and hydrogen bonding in **2** (atoms are shown as spheres of arbitrary size). Dashed lines represent hydrogen and halogen bonds. Only those atoms involved in halogen or hydrogen bonding are labeled. Symmetry codes: N12A = 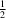 − *x*, *y* + 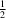, −*z* − 0.5; N21E/N22E = 1 − *x*, 2 − *y*, −1 − *z*; N22C = *x* − 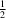, 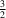 − *y*, *z* + 0.5; I15B = −*x*, 2 − *y*, −*z*.

**Figure 6:**
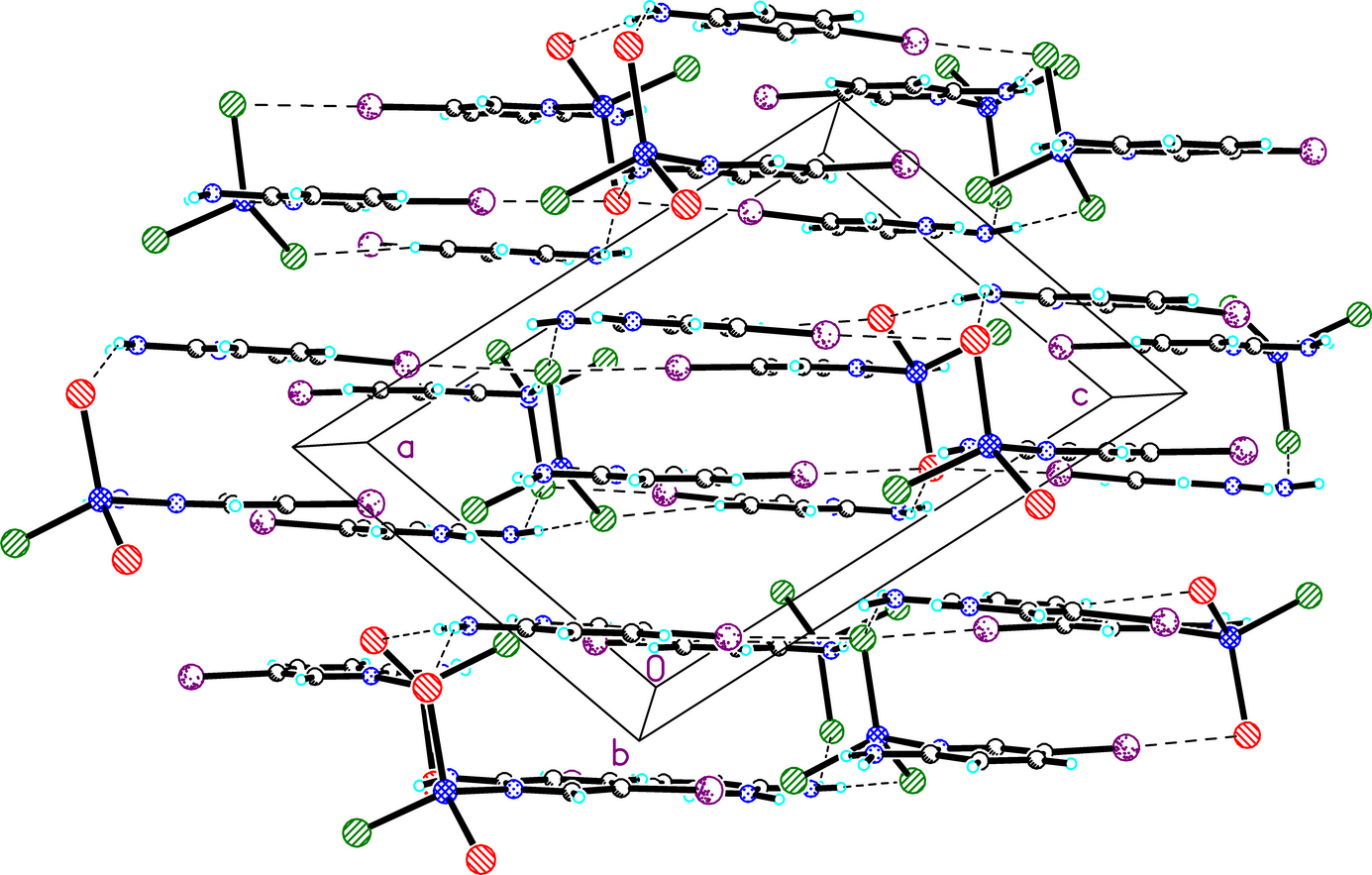
The structure of **2** viewed parallel to the *b* axis (atoms are shown as spheres of arbitrary size). Dashed lines represent hydrogen and halogen bonds.

Compound **2**. As with **1**, **2** exhibits multiple hydrogen (Table 3 ▸) and halogen bonds (Fig. 5 ▸). The hydrogen bonds are typical with *d*_D⋯*A*_ = 3.282 (6)–3.341 (10) Å and ∠_*D*—H⋯*A*_ = 151 (5)–176 (5)°. Type II halogen bonds are also observed between both the 5IAP ligand [*d*_I15⋯Cl1_ = 3.464 (3) Å, ∠_C15—I15⋯Cl1_ = 171.8 (3)°] and the 5IAPH cation [*d*_I25⋯Cl1_ = 3.511 (3) Å, ∠_C15—I15⋯Cl1_ = 175.5 (4)°. Unlike **1**, no I⋯I halogen bonds are observed. Similar to **1**, the structure forms layers of hydrogen and halogen bridged ions parallel to the *ac* face diagonal (Fig. 6 ▸). Unlike **1**, there are no direct linkages between those layers.

## 4. Database survey

The structures of a few salts of 5-IAPH have been reported. Copper(II) complexes include (5IAPH)[CuCl_3_(H_2_O)_2_]Cl (Abdalrahman *et al.*, 2013 ▸), two polymorphs of (5IAPH)_2_[CuCl_4_] (Giantsidis *et al.*, 2002 ▸) and (5IAPH)_2_[CuBr_4_]H_2_O (Landee *et al.*, 2001 ▸). Several Hg and Zn salts of 5IAPH have also been reported (Khavasi *et al.*, 2020 ▸), along with an Mn^II^ salt (Carnevale *et al.*, 2021 ▸). Copper complexes of 5IAP itself are also known including [(5IAP)_2_Cu*X*_2_], *X* = Cl, Br, [(5IAP)_2_CuBr_2_]_2_, [(5IAP)_3_CuCl_2_], (Huynh *et al.*, 2023 ▸) and [(5IAP)_2_CuBr(OMe)]_2_ (Araujo-Martinez *et al.*, 2023 ▸).

Compound **1** may be most conveniently compared to its corresponding hydrate and chloride analogue (Polson *et al.*, 2013 ▸). The C—N bond lengths in **1** are slightly shorter than observed in the hydrated salt (∼0.01–0.12 Å) and chloride analogue. Bond angles in **1** vary ±2° compared to the hydrated bromide salt, but not in any regular fashion, while they are comparable to those observed in the chloride complex within error.

With respect to compound **2**, although there no related compounds of 5-IAP, there are a number of reported structures including the [*LMX*_3_]^−^ ion where *L* is a pyridine-based ligand. Several of these involve Pt^II^ (Adams *et al.*, 2005 ▸; Bel’skii *et al.*, 1990 ▸; Rochon & Melanson, 1980 ▸; Melanson & Rochon, 1976 ▸) or Co^II^ (Bogdanovic *et al.*, 2001 ▸; Crane *et al.*, 2004 ▸). The closest comparisons appear to be compounds of Cu^II^ (Healy *et al.*, 1985 ▸; Savariault *et al.*, 1988 ▸) or Co^II^ (Hahn *et al.*, 1997 ▸) with the formulae (*L*H)[*LMX*_3_] (*L* = phenazine or quinoline for Cu, pyridine for Co). Similar hydrogen bonding is observed in all three compounds, but the absence of the iodine atom on the L group eliminates the halogen bonding observed in both **1** and **2**. Only in the quinolinium tri­chlorido­cuprate compound (Savariault *et al.*, 1988 ▸) are all of the aromatic rings approximately parallel, but even so the overall structure is that of dimers, rather than the extended sheet structure seen in **1** and **2**. With respect to the geometry at the metal ion, only the cobalt complex is similar with its slightly distorted tetra­hedral geometry (as compared to the two strongly Jahn–Teller-distorted Cu complexes) and slightly shorter Co—Cl bond lengths (average = 2.24 Å).

## 5. Synthesis and crystallization

Compound **1**: 2-Amino-5-iodo­pyridine (0.842g, 3.83 mmol) was dissolved in 10 mL of 9 *M* HBr and left to evaporate. After about one month, crystals of **1** were isolated by filtration (0.623g, 56%).

Compound **2**: HCl (0.0415 g, 12 *M*) and HBr (0.242 g. 9 *M*) were added to 2-amino-5-iodo­pyridine (0.439 g) creating a yellow solid. The solid was dissolved in 15 ml of 1-propanol and then cobalt(II) chloride hexa­hydrate (0.245 g) was added creating a dark-blue solution. After ten days, blue crystals were recovered by filtration. The crystals were predominantly lighter blue plates of (5IAPH)_2_[CoCl_4–*x*_Br_*x*_]·H_2_O (Mukda *et al.*, 2024 ▸), with a few dark-blue rhombic prisms mixed in. The dark-blue prisms were separated by hand and identified as compound **2** by X-ray diffraction.

## 6. Refinement

Crystal data, data collection and structure refinement details are summarized in Table 4 ▸. All non-hydrogen atoms were refined anisotropically. Hydrogen atoms bonded to carbon atoms were placed geometrically and refined with a riding model and *U*_iso_(H) = 1.2*U*_eq_(C). Hydrogen atoms bonded to nitro­gen atoms were located in a Fourier map and their positions refined with *U*_iso_(H) = 1.2*U*_eq_(N). Occupancies of the mixed halogen sites (*X*1 and *X*3) in **2** were allowed to refine freely. Mixed occupancy at the *X*2 site in **2** was initially assumed, but the bromide occupancy refined to zero within experimental error and the potential bromide ion was removed in the final refinement. Pseudo-isotropic restraints (ISOR) were applied to the lower occupancy ion, Br1.

## Supplementary Material

Crystal structure: contains datablock(s) 1, 2, publication_text. DOI: 10.1107/S2056989024010259/zn2039sup1.cif

Structure factors: contains datablock(s) 1. DOI: 10.1107/S2056989024010259/zn20391sup2.hkl

Structure factors: contains datablock(s) 2. DOI: 10.1107/S2056989024010259/zn20392sup3.hkl

CCDC references: 2392716, 2392715

Additional supporting information:  crystallographic information; 3D view; checkCIF report

## Figures and Tables

**Table 1:** Selected geometric parameters (Å, °) for **2**[Chem scheme1]

Co1—N11	2.034 (4)	Co1—Br1	2.328 (11)
Co1—Cl2	2.2648 (15)	Co1—Cl1	2.329 (6)
Co1—Cl3	2.306 (11)	Co1—Br3	2.342 (8)
			
N11—Co1—Cl2	111.63 (13)	N11—Co1—Cl1	105.87 (18)
N11—Co1—Cl3	105.1 (3)	Cl2—Co1—Cl1	109.17 (16)
Cl2—Co1—Cl3	108.0 (2)	Cl3—Co1—Cl1	116.9 (3)

**Table 2:** Hydrogen-bond geometry (Å, °) for **1**[Chem scheme1]

*D*—H⋯*A*	*D*—H	H⋯*A*	*D*⋯*A*	*D*—H⋯*A*
N11—H11⋯Br1	0.80 (2)	2.49 (2)	3.2136 (12)	150.8 (18)
N12—H12*A*⋯Br1^i^	0.86 (2)	2.55 (2)	3.3556 (12)	155.5 (17)
N12—H12*B*⋯Br1^ii^	0.85 (2)	2.67 (2)	3.4924 (13)	165.5 (18)

Symmetry codes: (i) 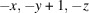; (ii) 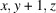.

**Table 3:** Hydrogen-bond geometry (Å, °) for **2**[Chem scheme1]

*D*—H⋯*A*	*D*—H	H⋯*A*	*D*⋯*A*	*D*—H⋯*A*
N12—H12*A*⋯Cl2	0.85 (6)	2.47 (7)	3.282 (6)	160 (5)
N12—H12*B*⋯Br1^i^	0.86 (7)	2.44 (7)	3.291 (12)	170 (6)
N22—H22*A*⋯Br1^ii^	0.87 (7)	2.55 (7)	3.338 (12)	151 (5)
N22—H22*B*⋯Br3^iii^	0.97 (6)	2.37 (7)	3.341 (10)	176 (5)

Symmetry codes: (i) 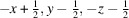; (ii) 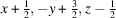; (iii) 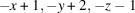.

**Table 4:** Experimental details

	**1**	**2**
Crystal data		
Chemical formula	C_5_H_6_IN_2_^+^·Br^−^	(C_5_H_6_IN_2_)[CoBr_0.51_Cl_2.48_(C_5_H_5_IN_2_)]
*M*_r_	300.93	629.20
Crystal system, space group	Triclinic, *P* 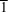	Monoclinic, *P*2_1_/*n*
Temperature (K)	100	100
*a*, *b*, *c* (Å)	5.2152 (2), 7.8039 (3), 10.1294 (4)	9.6998 (7), 13.5527 (8), 13.8518 (11)
α, β, γ (°)	93.3762 (12), 104.1108 (11), 96.4297 (12)	90, 107.336 (3), 90
*V* (Å^3^)	395.71 (3)	1738.2 (2)
*Z*	2	4
Radiation type	Mo *K*α	Mo *K*α
μ (mm^−1^)	9.01	6.10
Crystal size (mm)	0.24 × 0.21 × 0.08	0.11 × 0.07 × 0.06

Data collection		
Diffractometer	Bruker D8 Venture dual wavelength Mo/Cu	Bruker D8 Venture dual wavelength Mo/Cu
Absorption correction	Multi-scan (*SADABS*; Krause *et al.*, 2015 ▸)	Multi-scan (*SADABS*; Krause *et al.*, 2015 ▸)
*T*_min_, *T*_max_	0.605, 0.746	0.413, 0.492
No. of measured, independent and observed [*I* > 2σ(*I*)] reflections	13418, 2412, 2346	15915, 4318, 3139
*R*_int_	0.025	0.048
(sin θ/λ)_max_ (Å^−1^)	0.714	0.668

Refinement		
*R*[*F*^2^ > 2σ(*F*^2^)], *wR*(*F*^2^), *S*	0.012, 0.029, 1.11	0.040, 0.090, 1.09
No. of reflections	2412	4318
No. of parameters	91	216
No. of restraints	0	6
H-atom treatment	H atoms treated by a mixture of independent and constrained refinement	H atoms treated by a mixture of independent and constrained refinement
Δρ_max_, Δρ_min_ (e Å^−3^)	0.48, −0.50	1.22, −0.78

Computer programs: *APEX4* and *SAINT* (Bruker, 2022 ▸), *SHELXS2014* (Sheldrick 2008 ▸) and *SHELXL2019/2* (Sheldrick, 2015 ▸).

## supplementary crystallographic information

### 2-Amino-5-iodopyridinium bromide (1) Crystal data

**Table:**

C_5_H_6_IN_2_^+^·Br^−^	*Z* = 2
*M**_r_* = 300.93	*F*(000) = 276
Triclinic, *P*1	*D*_x_ = 2.526 Mg m^−^^3^
*a* = 5.2152 (2) Å	Mo *K*α radiation, λ = 0.71073 Å
*b* = 7.8039 (3) Å	Cell parameters from 9950 reflections
*c* = 10.1294 (4) Å	θ = 2.6–30.5°
α = 93.3762 (12)°	µ = 9.01 mm^−^^1^
β = 104.1108 (11)°	*T* = 100 K
γ = 96.4297 (12)°	Block, yellow
*V* = 395.71 (3) Å^3^	0.24 × 0.21 × 0.08 mm

### 2-Amino-5-iodopyridinium bromide (1) Data collection

**Table:**

Bruker D8 VENTURE dual wavelength Mo/Cu diffractometer	2412 independent reflections
Radiation source: microfocus sealed X-ray tube, Incoatec Iµs 3.0	2346 reflections with *I* > 2σ(*I*)
HELIOS double bounce multilayer mirror monochromator	*R*_int_ = 0.025
φ and ω scans	θ_max_ = 30.5°, θ_min_ = 2.1°
Absorption correction: multi-scan (SADABS; Krause *et al.*, 2015)	*h* = −7→7
*T*_min_ = 0.605, *T*_max_ = 0.746	*k* = −11→11
13418 measured reflections	*l* = −14→13

### 2-Amino-5-iodopyridinium bromide (1) Refinement

**Table:**

Refinement on *F*^2^	Primary atom site location: structure-invariant direct methods
Least-squares matrix: full	Secondary atom site location: difference Fourier map
*R*[*F*^2^ > 2σ(*F*^2^)] = 0.012	Hydrogen site location: mixed
*wR*(*F*^2^) = 0.029	H atoms treated by a mixture of independent and constrained refinement
*S* = 1.11	*w* = 1/[σ^2^(*F*_o_^2^) + 0.2575*P*] where *P* = (*F*_o_^2^ + 2*F*_c_^2^)/3
2412 reflections	(Δ/σ)_max_ = 0.002
91 parameters	Δρ_max_ = 0.48 e Å^−^^3^
0 restraints	Δρ_min_ = −0.50 e Å^−^^3^

### 2-Amino-5-iodopyridinium bromide (1) Special details

**Table:**

Experimental. Data collections for compounds 1 and 2 were carried out with a Bruker D8 Venture Photon III diffractometer employing Mo-Kα radiation (λ = 0.71073Å). The data were collected using Bruker Instrument Service v8.5.0.27 & APEX4 v2022.10-1 and reduced using Bruker SAINT v8.40b (Bruker, 2022). Absorption corrections were performed using SADABS (Krause, 2015). The structure was solved using SHELXS-2014 (Sheldrick, 2008) and refined using SHELXL-2019 (Sheldrick, 2015).
Geometry. All esds (except the esd in the dihedral angle between two l.s. planes) are estimated using the full covariance matrix. The cell esds are taken into account individually in the estimation of esds in distances, angles and torsion angles; correlations between esds in cell parameters are only used when they are defined by crystal symmetry. An approximate (isotropic) treatment of cell esds is used for estimating esds involving l.s. planes.

### 2-Amino-5-iodopyridinium bromide (1) Fractional atomic coordinates and isotropic or equivalent isotropic displacement parameters (Å^2^)

**Table:**

	*x*	*y*	*z*	*U*_iso_*/*U*_eq_	
N11	0.5947 (2)	0.68193 (15)	0.18178 (12)	0.0141 (2)	
H11	0.482 (4)	0.603 (3)	0.145 (2)	0.017*	
C12	0.5464 (3)	0.84605 (17)	0.16387 (13)	0.0128 (2)	
N12	0.3196 (2)	0.87844 (17)	0.08228 (13)	0.0164 (2)	
H12A	0.197 (4)	0.801 (3)	0.034 (2)	0.020*	
H12B	0.292 (4)	0.983 (3)	0.081 (2)	0.020*	
C13	0.7459 (3)	0.98060 (17)	0.23485 (14)	0.0141 (2)	
H13	0.722951	1.097926	0.221414	0.017*	
C14	0.9711 (3)	0.94205 (17)	0.32251 (14)	0.0146 (2)	
H14	1.103592	1.032609	0.371018	0.017*	
I15	1.33221 (2)	0.70519 (2)	0.48691 (2)	0.01678 (3)	
C15	1.0075 (3)	0.76766 (17)	0.34127 (13)	0.0140 (2)	
C16	0.8176 (3)	0.64035 (18)	0.26754 (14)	0.0149 (2)	
H16	0.841544	0.522265	0.276282	0.018*	
Br1	0.23627 (3)	0.31109 (2)	0.14445 (2)	0.01608 (4)	

### 2-Amino-5-iodopyridinium bromide (1) Atomic displacement parameters (Å^2^)

**Table:**

	*U*^11^	*U*^22^	*U*^33^	*U*^12^	*U*^13^	*U*^23^
N11	0.0168 (5)	0.0103 (5)	0.0141 (5)	−0.0016 (4)	0.0035 (4)	−0.0002 (4)
C12	0.0149 (6)	0.0121 (5)	0.0118 (5)	0.0003 (4)	0.0051 (4)	0.0005 (4)
N12	0.0150 (5)	0.0149 (5)	0.0171 (5)	0.0000 (4)	0.0008 (4)	0.0008 (4)
C13	0.0167 (6)	0.0106 (5)	0.0145 (6)	0.0010 (4)	0.0029 (5)	0.0020 (4)
C14	0.0149 (6)	0.0127 (6)	0.0147 (6)	−0.0005 (4)	0.0023 (5)	0.0007 (5)
I15	0.01575 (5)	0.01769 (5)	0.01789 (5)	0.00603 (3)	0.00364 (3)	0.00494 (3)
C15	0.0154 (6)	0.0144 (6)	0.0136 (6)	0.0035 (5)	0.0053 (5)	0.0033 (5)
C16	0.0193 (6)	0.0125 (6)	0.0145 (6)	0.0029 (5)	0.0063 (5)	0.0029 (5)
Br1	0.01538 (6)	0.01065 (6)	0.01964 (7)	−0.00109 (4)	0.00095 (5)	0.00055 (5)

### 2-Amino-5-iodopyridinium bromide (1) Geometric parameters (Å, º)

**Table:**

N11—C12	1.3469 (17)	C13—C14	1.3638 (19)
N11—C16	1.3553 (18)	C13—H13	0.9500
N11—H11	0.80 (2)	C14—C15	1.4125 (18)
C12—N12	1.3271 (18)	C14—H14	0.9500
C12—C13	1.4155 (18)	I15—C15	2.0815 (14)
N12—H12A	0.86 (2)	C15—C16	1.3628 (19)
N12—H12B	0.85 (2)	C16—H16	0.9500
			
C12—N11—C16	123.45 (12)	C12—C13—H13	120.0
C12—N11—H11	119.3 (14)	C13—C14—C15	120.09 (12)
C16—N11—H11	117.1 (14)	C13—C14—H14	120.0
N12—C12—N11	120.62 (13)	C15—C14—H14	120.0
N12—C12—C13	121.90 (12)	C16—C15—C14	118.68 (13)
N11—C12—C13	117.49 (12)	C16—C15—I15	120.29 (10)
C12—N12—H12A	124.8 (13)	C14—C15—I15	120.93 (10)
C12—N12—H12B	116.8 (14)	N11—C16—C15	120.10 (12)
H12A—N12—H12B	118.3 (19)	N11—C16—H16	119.9
C14—C13—C12	120.10 (12)	C15—C16—H16	119.9
C14—C13—H13	120.0		
			
C16—N11—C12—N12	−177.42 (13)	C13—C14—C15—C16	1.8 (2)
C16—N11—C12—C13	2.67 (19)	C13—C14—C15—I15	−174.50 (10)
N12—C12—C13—C14	177.04 (13)	C12—N11—C16—C15	0.0 (2)
N11—C12—C13—C14	−3.05 (19)	C14—C15—C16—N11	−2.25 (19)
C12—C13—C14—C15	0.9 (2)	I15—C15—C16—N11	174.05 (10)

### 2-Amino-5-iodopyridinium bromide (1) Hydrogen-bond geometry (Å, º)

**Table:**

*D*—H···*A*	*D*—H	H···*A*	*D*···*A*	*D*—H···*A*
N11—H11···Br1	0.80 (2)	2.49 (2)	3.2136 (12)	150.8 (18)
N12—H12*A*···Br1^i^	0.86 (2)	2.55 (2)	3.3556 (12)	155.5 (17)
N12—H12*B*···Br1^ii^	0.85 (2)	2.67 (2)	3.4924 (13)	165.5 (18)

Symmetry codes: (i) −*x*, −*y*+1, −*z*; (ii) *x*, *y*+1, *z*.

### 2-Amino-5-iodopyridinium (2-amino-5-iodopyridine-κN1)bromido/chlorido(0.51/2.48)cobalt(II) (2) Crystal data

**Table:**

(C_5_H_6_IN_2_)[CoBr_0.51_Cl_2.48_(C_5_H_5_IN_2_)]	*F*(000) = 1169
*M**_r_* = 629.20	*D*_x_ = 2.404 Mg m^−^^3^
Monoclinic, *P*2_1_/*n*	Mo *K*α radiation, λ = 0.71073 Å
*a* = 9.6998 (7) Å	Cell parameters from 5517 reflections
*b* = 13.5527 (8) Å	θ = 2.7–28.3°
*c* = 13.8518 (11) Å	µ = 6.10 mm^−^^1^
β = 107.336 (3)°	*T* = 100 K
*V* = 1738.2 (2) Å^3^	Block, blue
*Z* = 4	0.11 × 0.07 × 0.06 mm

### 2-Amino-5-iodopyridinium (2-amino-5-iodopyridine-κN1)bromido/chlorido(0.51/2.48)cobalt(II) (2) Data collection

**Table:**

Bruker D8 VENTURE dual wavelength Mo/Cu diffractometer	4318 independent reflections
Radiation source: microfocus sealed X-ray tube, Incoatec Iµs 3.0	3139 reflections with *I* > 2σ(*I*)
HELIOS double bounce multilayer mirror monochromator	*R*_int_ = 0.048
φ and ω scans	θ_max_ = 28.3°, θ_min_ = 2.2°
Absorption correction: multi-scan (SADABS; Krause *et al.*, 2015)	*h* = −12→10
*T*_min_ = 0.413, *T*_max_ = 0.492	*k* = −18→16
15915 measured reflections	*l* = −14→18

### 2-Amino-5-iodopyridinium (2-amino-5-iodopyridine-κN1)bromido/chlorido(0.51/2.48)cobalt(II) (2) Refinement

**Table:**

Refinement on *F*^2^	Primary atom site location: structure-invariant direct methods
Least-squares matrix: full	Secondary atom site location: difference Fourier map
*R*[*F*^2^ > 2σ(*F*^2^)] = 0.040	Hydrogen site location: mixed
*wR*(*F*^2^) = 0.090	H atoms treated by a mixture of independent and constrained refinement
*S* = 1.09	*w* = 1/[σ^2^(*F*_o_^2^) + (0.0334*P*)^2^ + 3.1822*P*] where *P* = (*F*_o_^2^ + 2*F*_c_^2^)/3
4318 reflections	(Δ/σ)_max_ = 0.001
216 parameters	Δρ_max_ = 1.22 e Å^−^^3^
6 restraints	Δρ_min_ = −0.77 e Å^−^^3^

### 2-Amino-5-iodopyridinium (2-amino-5-iodopyridine-κN1)bromido/chlorido(0.51/2.48)cobalt(II) (2) Special details

**Table:**

Geometry. All esds (except the esd in the dihedral angle between two l.s. planes) are estimated using the full covariance matrix. The cell esds are taken into account individually in the estimation of esds in distances, angles and torsion angles; correlations between esds in cell parameters are only used when they are defined by crystal symmetry. An approximate (isotropic) treatment of cell esds is used for estimating esds involving l.s. planes.

### 2-Amino-5-iodopyridinium (2-amino-5-iodopyridine-κN1)bromido/chlorido(0.51/2.48)cobalt(II) (2) Fractional atomic coordinates and isotropic or equivalent isotropic displacement parameters (Å^2^)

**Table:**

	*x*	*y*	*z*	*U*_iso_*/*U*_eq_	Occ. (<1)
Co1	0.13812 (7)	0.97174 (5)	−0.26955 (6)	0.01941 (17)	
Br1	0.3650 (11)	1.0006 (8)	−0.1556 (9)	0.034 (4)	0.203 (5)
Cl1	0.3649 (7)	0.9995 (3)	−0.1548 (5)	0.0174 (19)	0.797 (5)
Cl2	0.16479 (13)	0.92952 (9)	−0.42118 (10)	0.0202 (3)	
Br3	−0.0247 (7)	1.1037 (7)	−0.2947 (4)	0.0155 (17)	0.311 (6)
Cl3	−0.0261 (11)	1.0994 (9)	−0.2955 (7)	0.039 (4)	0.689 (6)
N11	0.0486 (4)	0.8604 (3)	−0.2101 (3)	0.0177 (9)	
N12	0.1448 (5)	0.7359 (4)	−0.2837 (4)	0.0255 (11)	
H12A	0.170 (6)	0.779 (5)	−0.319 (5)	0.031*	
H12B	0.146 (7)	0.677 (5)	−0.305 (5)	0.031*	
C12	0.0668 (5)	0.7640 (4)	−0.2239 (4)	0.0205 (11)	
C13	0.0025 (6)	0.6924 (4)	−0.1763 (4)	0.0250 (13)	
H13	0.012753	0.624131	−0.188153	0.030*	
C14	−0.0742 (5)	0.7219 (4)	−0.1134 (4)	0.0245 (12)	
H14	−0.116299	0.674373	−0.080308	0.029*	
I15	−0.19969 (4)	0.87761 (3)	−0.00022 (3)	0.02480 (11)	
C15	−0.0903 (5)	0.8235 (4)	−0.0978 (4)	0.0221 (12)	
C16	−0.0286 (5)	0.8888 (4)	−0.1475 (4)	0.0218 (12)	
H16	−0.040037	0.957340	−0.137836	0.026*	
N21	0.8337 (5)	0.8524 (3)	−0.4898 (4)	0.0220 (10)	
H21	0.882 (6)	0.896 (4)	−0.522 (4)	0.026*	
C22	0.8517 (5)	0.7548 (4)	−0.5034 (4)	0.0206 (11)	
N22	0.9307 (5)	0.7271 (4)	−0.5612 (4)	0.0280 (11)	
H22A	0.946 (7)	0.665 (5)	−0.571 (5)	0.034*	
H22B	0.962 (6)	0.775 (5)	−0.602 (5)	0.034*	
C23	0.7858 (5)	0.6896 (4)	−0.4513 (4)	0.0223 (12)	
H23	0.796975	0.620461	−0.457159	0.027*	
C24	0.7061 (5)	0.7247 (4)	−0.3925 (4)	0.0215 (12)	
H24	0.662676	0.680358	−0.357108	0.026*	
C25	0.6888 (5)	0.8281 (4)	−0.3847 (4)	0.0220 (12)	
I25	0.56035 (4)	0.88657 (3)	−0.30094 (3)	0.02550 (11)	
C26	0.7535 (5)	0.8891 (4)	−0.4331 (4)	0.0202 (11)	
H26	0.743268	0.958429	−0.427857	0.024*	

### 2-Amino-5-iodopyridinium (2-amino-5-iodopyridine-κN1)bromido/chlorido(0.51/2.48)cobalt(II) (2) Atomic displacement parameters (Å^2^)

**Table:**

	*U*^11^	*U*^22^	*U*^33^	*U*^12^	*U*^13^	*U*^23^
Co1	0.0173 (3)	0.0176 (4)	0.0231 (4)	−0.0007 (3)	0.0057 (3)	0.0006 (3)
Br1	0.011 (5)	0.054 (6)	0.042 (8)	0.000 (4)	0.014 (4)	0.006 (5)
Cl1	0.026 (4)	0.0045 (18)	0.021 (3)	0.0005 (17)	0.006 (2)	−0.0007 (17)
Cl2	0.0173 (5)	0.0213 (7)	0.0214 (7)	0.0003 (5)	0.0050 (5)	0.0009 (5)
Br3	0.017 (3)	0.020 (3)	0.009 (2)	0.004 (2)	0.0037 (19)	0.002 (2)
Cl3	0.047 (6)	0.019 (4)	0.053 (6)	0.000 (3)	0.018 (4)	−0.001 (3)
N11	0.0156 (19)	0.015 (2)	0.021 (2)	−0.0005 (17)	0.0038 (17)	0.0003 (18)
N12	0.035 (3)	0.019 (3)	0.023 (3)	0.002 (2)	0.010 (2)	−0.002 (2)
C12	0.016 (2)	0.018 (3)	0.024 (3)	0.002 (2)	0.002 (2)	−0.005 (2)
C13	0.023 (3)	0.021 (3)	0.025 (3)	−0.001 (2)	−0.001 (2)	−0.003 (2)
C14	0.018 (2)	0.026 (3)	0.024 (3)	−0.008 (2)	−0.001 (2)	0.005 (2)
I15	0.02164 (18)	0.0306 (2)	0.0235 (2)	−0.00515 (15)	0.00868 (14)	−0.00071 (16)
C15	0.015 (2)	0.024 (3)	0.025 (3)	−0.003 (2)	0.002 (2)	−0.003 (2)
C16	0.019 (2)	0.020 (3)	0.024 (3)	−0.001 (2)	0.002 (2)	−0.002 (2)
N21	0.021 (2)	0.020 (2)	0.025 (3)	−0.0046 (19)	0.0050 (19)	−0.0008 (19)
C22	0.015 (2)	0.021 (3)	0.024 (3)	0.000 (2)	0.002 (2)	−0.003 (2)
N22	0.025 (2)	0.023 (3)	0.038 (3)	0.000 (2)	0.014 (2)	−0.003 (2)
C23	0.020 (2)	0.017 (3)	0.028 (3)	−0.003 (2)	0.003 (2)	0.001 (2)
C24	0.016 (2)	0.021 (3)	0.025 (3)	−0.004 (2)	0.003 (2)	0.003 (2)
C25	0.013 (2)	0.030 (3)	0.018 (3)	−0.001 (2)	−0.002 (2)	−0.004 (2)
I25	0.01950 (17)	0.0274 (2)	0.0308 (2)	0.00454 (15)	0.00938 (15)	0.00033 (16)
C26	0.018 (2)	0.017 (3)	0.025 (3)	0.003 (2)	0.005 (2)	−0.004 (2)

### 2-Amino-5-iodopyridinium (2-amino-5-iodopyridine-κN1)bromido/chlorido(0.51/2.48)cobalt(II) (2) Geometric parameters (Å, º)

**Table:**

Co1—N11	2.034 (4)	C15—C16	1.364 (8)
Co1—Cl2	2.2648 (15)	C16—H16	0.9500
Co1—Cl3	2.306 (11)	N21—C26	1.354 (7)
Co1—Br1	2.328 (11)	N21—C22	1.354 (7)
Co1—Cl1	2.329 (6)	N21—H21	0.95 (6)
Co1—Br3	2.342 (8)	C22—N22	1.318 (7)
N11—C12	1.339 (6)	C22—C23	1.409 (8)
N11—C16	1.359 (7)	N22—H22A	0.87 (7)
N12—C12	1.333 (7)	N22—H22B	0.97 (6)
N12—H12A	0.85 (6)	C23—C24	1.364 (8)
N12—H12B	0.86 (7)	C23—H23	0.9500
C12—C13	1.418 (8)	C24—C25	1.420 (8)
C13—C14	1.363 (8)	C24—H24	0.9500
C13—H13	0.9500	C25—C26	1.333 (8)
C14—C15	1.410 (8)	C25—I25	2.095 (6)
C14—H14	0.9500	C26—H26	0.9500
I15—C15	2.084 (6)		
			
N11—Co1—Cl2	111.63 (13)	C16—C15—I15	118.9 (4)
N11—Co1—Cl3	105.1 (3)	C14—C15—I15	123.0 (4)
Cl2—Co1—Cl3	108.0 (2)	N11—C16—C15	123.1 (5)
N11—Co1—Br1	106.4 (3)	N11—C16—H16	118.4
Cl2—Co1—Br1	109.0 (3)	C15—C16—H16	118.4
N11—Co1—Cl1	105.87 (18)	C26—N21—C22	124.0 (5)
Cl2—Co1—Cl1	109.17 (16)	C26—N21—H21	120 (4)
Cl3—Co1—Cl1	116.9 (3)	C22—N21—H21	116 (4)
N11—Co1—Br3	106.1 (2)	N22—C22—N21	119.1 (5)
Cl2—Co1—Br3	108.25 (15)	N22—C22—C23	124.5 (5)
Br1—Co1—Br3	115.6 (3)	N21—C22—C23	116.4 (5)
C12—N11—C16	119.2 (5)	C22—N22—H22A	122 (4)
C12—N11—Co1	125.2 (4)	C22—N22—H22B	121 (4)
C16—N11—Co1	115.5 (3)	H22A—N22—H22B	117 (6)
C12—N12—H12A	119 (4)	C24—C23—C22	120.8 (5)
C12—N12—H12B	124 (4)	C24—C23—H23	119.6
H12A—N12—H12B	114 (6)	C22—C23—H23	119.6
N12—C12—N11	119.4 (5)	C23—C24—C25	119.4 (5)
N12—C12—C13	120.2 (5)	C23—C24—H24	120.3
N11—C12—C13	120.4 (5)	C25—C24—H24	120.3
C14—C13—C12	119.7 (5)	C26—C25—C24	119.3 (5)
C14—C13—H13	120.1	C26—C25—I25	119.4 (4)
C12—C13—H13	120.1	C24—C25—I25	121.2 (4)
C13—C14—C15	119.4 (5)	C25—C26—N21	120.1 (5)
C13—C14—H14	120.3	C25—C26—H26	119.9
C15—C14—H14	120.3	N21—C26—H26	119.9
C16—C15—C14	118.1 (5)		
			
C16—N11—C12—N12	−178.7 (5)	I15—C15—C16—N11	177.9 (4)
Co1—N11—C12—N12	−2.5 (7)	C26—N21—C22—N22	179.2 (5)
C16—N11—C12—C13	1.9 (7)	C26—N21—C22—C23	−2.4 (7)
Co1—N11—C12—C13	178.2 (4)	N22—C22—C23—C24	179.7 (5)
N12—C12—C13—C14	178.4 (5)	N21—C22—C23—C24	1.3 (7)
N11—C12—C13—C14	−2.2 (8)	C22—C23—C24—C25	0.5 (8)
C12—C13—C14—C15	1.0 (8)	C23—C24—C25—C26	−1.5 (8)
C13—C14—C15—C16	0.4 (8)	C23—C24—C25—I25	177.2 (4)
C13—C14—C15—I15	−178.1 (4)	C24—C25—C26—N21	0.6 (8)
C12—N11—C16—C15	−0.5 (8)	I25—C25—C26—N21	−178.2 (4)
Co1—N11—C16—C15	−177.1 (4)	C22—N21—C26—C25	1.5 (8)
C14—C15—C16—N11	−0.7 (8)		

### 2-Amino-5-iodopyridinium (2-amino-5-iodopyridine-κN1)bromido/chlorido(0.51/2.48)cobalt(II) (2) Hydrogen-bond geometry (Å, º)

**Table:**

*D*—H···*A*	*D*—H	H···*A*	*D*···*A*	*D*—H···*A*
N12—H12*A*···Cl2	0.85 (6)	2.47 (7)	3.282 (6)	160 (5)
N12—H12*B*···Br1^i^	0.86 (7)	2.44 (7)	3.291 (12)	170 (6)
N22—H22*A*···Br1^ii^	0.87 (7)	2.55 (7)	3.338 (12)	151 (5)
N22—H22*A*···I15^iii^	0.87 (7)	3.33 (6)	3.711 (5)	110 (5)
N22—H22*B*···Br3^iv^	0.97 (6)	2.37 (7)	3.341 (10)	176 (5)

Symmetry codes: (i) −*x*+1/2, *y*−1/2, −*z*−1/2; (ii) *x*+1/2, −*y*+3/2, *z*−1/2; (iii) *x*+3/2, −*y*+3/2, *z*−1/2; (iv) −*x*+1, −*y*+2, −*z*−1.

## References

[bb1] Abdalrahman, M. A., Awwadi, F. F., Jameson, G. B., Landee, C. P., Saunders, C. G., Turnbull, M. M. & Wikaira, J. L. (2013). *CrystEngComm*, **15**, 4309–4320.

[bb2] Adams, C. J., Crawford, P. C., Orpen, A. G., Podesta, T. J. & Salt, B. (2005). *Chem. Commun.* pp. 2457–2458. 10.1039/b501555c 15886769

[bb3] Anderson, P. W. (1958). *Phys. Rev.* **109**, 1492–1505.

[bb4] Araujo-Martinez, A. P., Faber, K., Huynh, N., Dickie, D. A., Landee, C. P., Turnbull, M. M., Twamley, B. & Wikaira, J. L. (2023). *J. Coord. Chem.* **76**, 632–647.

[bb5] Bel’skii, V. K., Kukushkin, V. Yu., Konovalov, V. E., Moiseev, A. I. & Yakovlev, V. N. (1990). *Zh. Obshch. Khim.* **60**, 2180–2189.

[bb6] Bogdanović, G. A., Medaković, V. B., Vojinović, L. S., Češljević, V. I., Leovac, V. M., Spasojević-de Biré, A. & Zarić, S. D. (2001). *Polyhedron*, **20**, 2231–2240.

[bb7] Bruker (2022). *APEX4* and *SAINT*. Bruker AXS Inc., Madison, Wisconsin, USA.

[bb8] Carnevale, D. J., Dawe, L. N., Landee, C. P., Turnbull, M. M. & Wikaira, J. L. (2021). *Polyhedron*, **202**, 115200.

[bb9] Coey, J. M. D. (1978). *J. Appl. Phys.* **49**, 1646–1652.

[bb10] Coey, J. M. D., Venkatesan, M. & Fitzgerald, C. B. (2005). *Nat. Mater.* **4**, 173–179. 10.1038/nmat1310 15654343

[bb11] Coffey, T. J., Landee, C. P., Robinson, W. T., Turnbull, M. M., Winn, M. & Woodward, F. M. (2000). *Inorg. Chim. Acta*, **303**, 54–60.

[bb12] Crane, J. D., Emeleus, L. C., Harrison, D. & Nilsson, P. A. (2004). *Inorg. Chim. Acta*, **357**, 3407–3412.

[bb13] Fujiwara, N., Jeitler, J. R., Navas, C., Turnbull, M. M., Goto, T. & Hosoito, N. (1995). *J. Magn. Magn. Mater.* **140–144**, 1663–1664.

[bb14] Giantsidis, J., Galeriu, C., Landee, C. P. & Turnbull, M. M. (2002). *J. Coord. Chem.* **55**, 795–803.

[bb15] Hahn, F. E., Scharn, D. & Lügger, T. (1997). *Z. Kristallogr. New Cryst. Struct.* **212**, 472–472.

[bb16] Healy, P. C., Pakawatchai, C. & White, A. H. (1985). *Aust. J. Chem.* **38**, 669–675.

[bb17] Huynh, N. V., Li, L., Landee, C. P., Dawe, L. N., Dickie, D. A., Turnbull, M. M. & Wikaira, J. L. (2023). *Polyhedron*, **243**, 116562.

[bb18] Khavasi, H. R., Gholami, A., Hosseini, M., Nikpoor, L. & Eskandari, K. (2020). *Cryst. Growth Des.* **20**, 2266–2274.

[bb19] Khrennikov, A. (2016). *Int. J. Quantum Information*, **14**, 1640009.

[bb20] Kimchi, I., Nahum, A. & Senthil, T. (2018). *Phys. Rev. X*, **8**, 0310281.

[bb21] Krause, L., Herbst-Irmer, R., Sheldrick, G. M. & Stalke, D. (2015). *J. Appl. Cryst.* **48**, 3–10. 10.1107/S1600576714022985 PMC4453166 26089746

[bb22] Landee, C. P., Turnbull, M. M., Galeriu, C., Giantsidis, J. & Woodward, F. M. (2001). *Phys. Rev. B*, **63**, 100402R.

[bb23] Mackenzie, J. K. (1964). *Acta Metall.* **12**, 223–225.

[bb24] Melanson, R. & Rochon, F. D. (1976). *Can. J. Chem.* **54**, 1002–1006.

[bb25] Mukda, B. A., Giantsidis, J., Landee, C. P., Dickie, D. A., Wikaira, J. L. & Turnbull, M. M. (2024). *J. Coord. Chem.* Submitted.

[bb26] Polson, M., Turnbull, M. M. & Wikaira, J. L. (2013). *Acta Cryst.* C**69**, 1152–1156. 10.1107/S0108270113020891 24096506

[bb27] Rochon, F. D. & Melanson, R. (1980). *Acta Cryst.* B**36**, 691–693.

[bb28] Savariault, J. M., Galy, J., Gutierrez-Zorrilla, J. M. & Roman, P. (1988). *J. Mol. Struct.* **176**, 313–322.

[bb29] Sheldrick, G. M. (2008). *Acta Cryst.* A**64**, 112–122. 10.1107/S0108767307043930 18156677

[bb30] Sheldrick, G. M. (2015). *Acta Cryst.* C**71**, 3–8.

[bb31] Toulouse, G. (1986). In *Spin Glass Theory and Beyond.* World Scientific Lecture Notes in Physics, edited by M. Mezard, G. Parisi & M. Virasoro, Vol. 9, pp 99–103. Singapore: World Scientific.

[bb32] Woodward, F. M., Albrecht, A. S., Wynn, C. M., Landee, C. P. & Turnbull, M. M. (2002). *Phys. Rev. B*, **65**, 144412.

